# Development and validation of the CHIRTS-daily quasi-global high-resolution daily temperature data set

**DOI:** 10.1038/s41597-020-00643-7

**Published:** 2020-09-14

**Authors:** Andrew Verdin, Chris Funk, Pete Peterson, Martin Landsfeld, Cascade Tuholske, Kathryn Grace

**Affiliations:** 1 Minnesota Population Center, University of Minnesota Twin Cities, Minnesota, USA; 2 U.S. Geological Survey, Earth Resources Observation and Science (EROS) Center, Sioux Falls, South Dakota USA; 3 Climate Hazards Center, University of California, Santa Barbara, USA; 4 Department of Geography, University of California, Santa Barbara, USA; 5 Department of Geography, Environment and Society, University of Minnesota Twin Cities, Minnesota, USA

**Keywords:** Geography, Scientific data

## Abstract

We present a high-resolution daily temperature data set, CHIRTS-daily, which is derived by merging the monthly Climate Hazards center InfraRed Temperature with Stations climate record with daily temperatures from version 5 of the European Centre for Medium-Range Weather Forecasts Re-Analysis. We demonstrate that remotely sensed temperature estimates may more closely represent true conditions than those that rely on interpolation, especially in regions with sparse *in situ* data. By leveraging remotely sensed infrared temperature observations, CHIRTS-daily provides estimates of 2-meter air temperature for 1983–2016 with a footprint covering 60°S-70°N. We describe this data set and perform a series of validations using station observations from two prominent climate data sources. The validations indicate high levels of accuracy, with CHIRTS-daily correlations with observations ranging from 0.7 to 0.9, and very good representation of heat wave trends.

## Abstract (summary)

**Table:** 

Measurement(s)	temperature of air
Technology Type(s)	infrared radiation
Sample Characteristic - Environment	atmospheric layer
Sample Characteristic - Location	Africa • Australia • Eurasia • North America • South America

Machine-accessible metadata file describing the reported data: 10.6084/m9.figshare.12593846

## Background & Summary

**Fig. 1:**
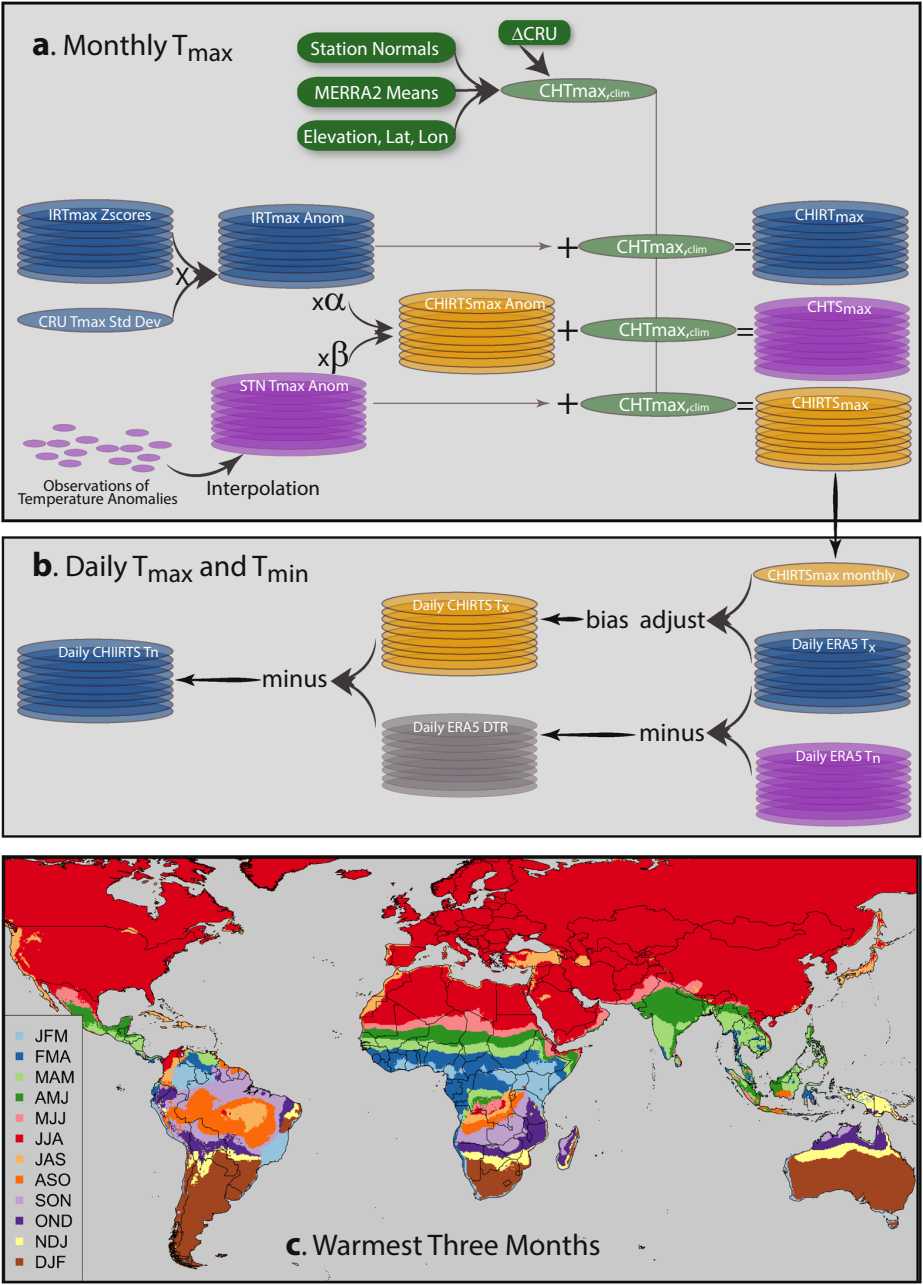
Description of the CHIRTS-daily development and validation processes. (**a)** Description of the CHIRTS_max_ monthly T_max_ development and validation processes. (**b**) Schema of CHIRTS-daily T_max_ (T_x_) and T_min_ (T_n_) development process. (**c**) Hottest three consecutive months derived from CHT_clim_ (CHIRTS_max_ climatology).

This manuscript is focused on the development and validation of CHIRTS-daily: a high-resolution (0.05° × 0.05°) daily maximum and minimum temperature data series spanning 60°S–70°N. The CHIRTS-daily data set builds on the Climate Hazards center InfraRed Temperature with Stations T_max_ data set (CHIRTS_max_^1^) (Fig. 1a), which leverages remotely sensed infrared land surface emission temperatures and a dense global network of approximately 15,000 Berkeley Earth *in situ* station observations to provide robust high-resolution (0.05° × 0.05°) estimates of monthly mean maximum 2-meter air temperature (T_max_). The CHIRTS_max_ product focuses only on maximum air temperature estimates because minimum temperatures are more difficult to distinguish from the cool cloud-top temperatures observed by satellites. For T_max_ values, cloud screening can be used to isolate the thermal infrared land surface temperature signal, which correlates well with the monthly maximum 2-meter temperatures observed at weather stations. To support scientific modeling, as well as health, agriculture, and ecological applications that often demand finer temporal resolution, we have created a daily CHIRTS product (CHIRTS-daily) by using daily T_max_ and T_min_ fields from version 5 of the European Centre for Medium-Range Weather Forecasts Re-Analysis (ERA5; https://cds.climate.copernicus.eu/cdsapp#!/home) to disaggregate the monthly CHIRTS_max_ product. The ERA5 data set is an advanced reanalysis that uses weather and land models, forced with satellite and *in situ* observations, to derive a complete suite of physically consistent variables describing many aspects of the Earth. The daily ERA5 diurnal temperature range (DTR; defined as maximum temperature minus minimum temperature) is then used to create CHIRTS-daily minimum temperatures. The ERA5 DTR values are based on the hourly reanalysis simulations, which incorporate physically based land and atmospheric modeling components, and hence are not affected by potential cloud contamination. Figure 1b provides a graphical representation of the CHIRTS-daily development process. The monthly CHIRTS_max_ is built around an accurate very high resolution climatology, thereby providing important spatial detail. The month-to-month variations in CHIRTS_max_ leverage the power of satellite observations to provide skillful estimates in data-sparse regions. Within any given month, the dynamically consistent ERA5 provides accurate assessments of day-to-day temperature anomalies. This approach uses the monthly CHIRTS_max_ to provide a reliable and consistent representation of the climate, and the daily ERA5 to represent weather.

Validation of the CHIRTS-daily maximum (CHIRTS_X_) and minimum (CHIRTS_N_) temperature products is carried out using daily climate station observations collected from the Global Historical Climatology Network (GHCN^2^) and the Global Summary of the Day (GSOD; https://data.noaa.gov/dataset/dataset/global-surface-summary-of-the-day-gsod/). We focus on validating in anomaly space to reduce the influence of the seasonal cycle on validation statistics. However, a complementary comparison of mean bias is also presented. This series of validations is limited to the three hottest consecutive months (Fig. 1c), identified using long-term monthly CHIRTS_max_ averages. We focus on the three hottest consecutive months under the assumption that many users will be interested in performance during the hottest times of the year. For each GHCN and GSOD station, CHIRTS_X_ and CHIRTS_N_ estimates for the nearest pixel are extracted and validation measures computed.

**Fig. 2:**
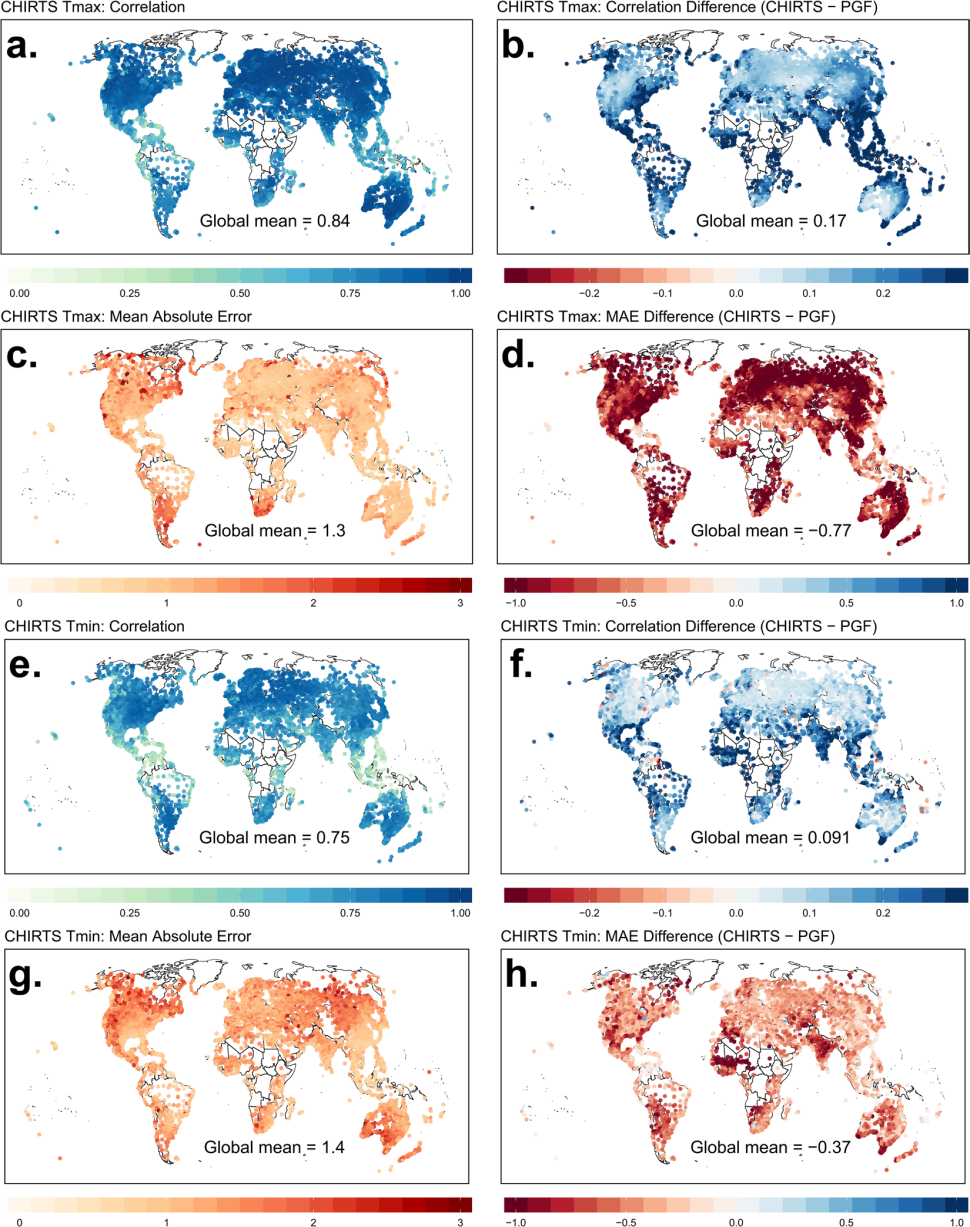
Validation statistics for maximum and minimum temperatures and a comparison between CHIRTS and PGF. (**a**) Correlation between CHIRTS_X_ and validation data sets. (**b**) Difference between correlations for CHIRTS_X_ and PGF_X_. **c**. Mean absolute error of CHIRTS_X_ with respect to validation data sets. (**d**) Difference between mean absolute errors for CHIRTS_X_ and PGF_X_. (**e**) Correlation between CHIRTS_N_ and validation data sets. (**f**) Difference between correlations for CHIRTS_N_ and PGF_N_. (**g**) Mean absolute error of CHIRTS_N_ with respect to validation data sets. (**h**) Difference between mean absolute errors for CHIRTS_N_ and PGF_N_.

**Fig. 3:**
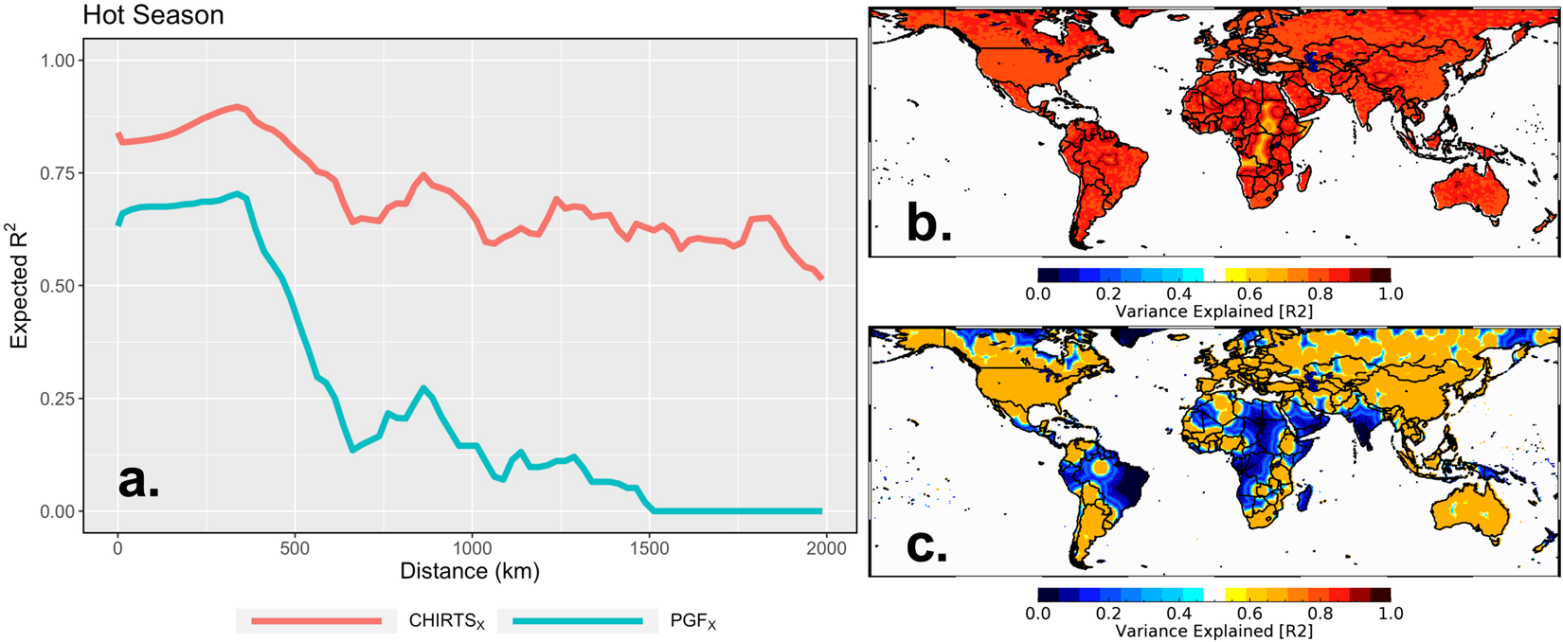
Performance of CHIRTS_X_ and PGF_X_, expressed as variance explained (R^2^) as a function of distance from the nearest CRU station, for the hottest three months. (**a**) Expected R^2^ as a function of distance from the grid cell to the nearest station. (**b**) Expected R^2^ of CHIRTS_X_ based on distribution of Berkeley Earth stations for January 2016. (**c**) Expected R^2^ of PGF_X_ based on distribution of CRU stations for January 2016.

The performance of CHIRTS_X_ and CHIRTS_N_ is compared to minimum and maximum temperatures from the Princeton Global Forcing (PGF^3^) data set, which is produced by the Terrestrial Hydrology Group at Princeton University. We find that CHIRTS-daily exhibits greater skill in capturing temperature anomalies, most notably in data-sparse regions (Fig. 2). As discussed at length in the CHIRTS_max_ manuscript^1^, the number of global *in situ* station observations is low and in decline—in fact, much of Earth is a data-sparse region (Fig. 3).

Spatial covariograms (Fig. 3a) quantify the performance of both products as a function of distance from the nearest station used in the gridded data set from the Climatic Research Unit at the University of East Anglia (CRU^4^). The x-axis of this graph shows binned distance values, and the vertical axis displays variance explained (R^2^) values. Performance (R^2^) for both products decays as the distance from the nearest CRU station increases, but this decay is much less in the CHIRTS-daily product. We use the CRU station archive because it is generally considered to be the gold standard, and because PGF is bias corrected using the monthly CRU. Subsequently, we produce maps of expected performance based on each product’s station density for January 2016 (Fig. 3b,c). These maps are based on the empirical covariograms shown in Fig. 3a. CHIRTS-daily is bolstered by not only the ~15,000 monthly Berkeley Earth station observations, but also the thermal infrared backbone from the monthly CHIRTS_max_, which better captures temperature anomalies in data-sparse regions. Areas where CHIRTS-daily begins to have relatively poor performance include a relatively large swath across central Africa, the Horn of Africa, and a small bubble in northern Mali. However, relatively poor performance for CHIRTS-daily indicates an expected R^2^ of about 0.60, whereas the expected performance of PGF in these same regions is closer to an expected R^2^ of 0.20.

## Methods

### Motivation

Assessing weather-related hazards in a changing climate requires data sets that have accurate high-resolution spatial mean fields, good performance in data-sparse regions, and limited sources of non-stationary errors. Many scientists are interested in the impacts associated with a degree or two of warming. But non-stationary errors—errors that create time-varying biases in data sets—can easily be as large or larger than this climate change signal. Spatial fidelity also matters. High-resolution mean fields are important because impacts to health, agriculture, and other sectors are always local and typically non-linear. Impacts on humans or crops will be related to extremes in specific locations, and these impacts will often be strongly related to the variations in the absolute value of the weather variable under consideration. A 1- or 2-degree change in mean temperature, for example, can dramatically alter the number of days exceeding some specific temperature threshold. This spatial accuracy is important for monitoring extreme events on a year-to-year basis, and for assessing the impacts of climate change. As demonstrated below, data sets that are too cool may also underestimate changes in the frequency of heat waves.

Another important consideration is performance in areas with low densities of publicly available weather stations. Climate hazards typically convolve weather-related shocks, human exposure, and human vulnerability. The most vulnerable populations, and those with the most rapidly expanding populations and exposure, are often in areas (like Africa, Central America, and parts of Asia) with few available *in situ* weather observations.

A third consideration is consistency and stationarity, both of which can be important in data-sparse areas, where the spatial location and density of available *in situ* observations changes over time, and typically declines. When weather station observations are blended with spatially explicit background fields, non-stationary systematic errors can arise, either through changes in the observational network and/or discontinuities in the spatially explicit background fields. Accurate high-resolution mean fields can reduce homogeneities arising from shifts in observational networks. When the spatially explicit background fields track closely with the *in situ* observations, disruptions associated with changing networks are minimized. Spurious systematic non-stationary errors in the background fields, however, can create large incorrect changes.

In general, there are two main approaches to overcoming these limitations: the creation of tailored data sets that combine satellite proxy information with weather station observations, or alternately, the use of ready-made modern reanalysis systems.

Drought early warning systems, especially those focused on data-sparse regions, must grapple with these issues. While the accurate and early identification of drought conditions can trigger mitigation activities that save lives and livelihoods, consistent, accurate, high resolution data sets are required to make such assessments. For 20 years, scientists at the University of California, Santa Barbara’s (UCSB) Climate Hazards Center (CHC) have focused on developing high-quality precipitation estimates suitable for supporting famine early warning, and crop and hydrologic modeling in data-sparse regions. The result of this work, the Climate Hazards center InfraRed Precipitation with Stations (CHIRPS^5^) is now one of the most widely used products for global drought monitoring. CHIRPS has been adopted by the World Food Program, the US Agency for International Development’s (USAID) Famine Early Warning Systems Network (FEWS NET, www.fews.net), the European Union, the Food and Agricultural Organization (FAO), and a host of regional and national agencies. The spatial resolution, accuracy, and consistency of CHIRPS also make it widely useful for applications such as crop insurance and climate change studies. In a typical month, more than 700 unique users download more than 100 gigabytes of CHIRPS data. The CHIRPS data set is hosted at the CHC, whose large computational capacity arises from ongoing support for and by FEWS NET. Every month, CHIRPS, along with many other valuable environmental data sets, helps FEWS NET guide billions of humanitarian assistance dollars to millions of extremely food-insecure people.

At present, there is a dearth of accurate information supporting the monitoring and evaluation of extreme temperatures in many food-insecure regions. Such extremes can wilt crops or decimate livestock herds, setting the stage for famine. Yet our ability to track these extremes in countries without weather station observations remains limited.

To address this limitation, the CHC has developed a modeling philosophy based on a geostatistical framework that decomposes environmental variables into static mean fields and time-varying anomaly fields. There are two stages to this process: a monthly T_max_ algorithm, described in the CHIRTS_max_ manuscript^1^ and illustrated in Fig. 1a, and a daily disaggregation procedure, described in Fig. 1b and in the following sections. In the CHC’s approach, great attention is given to building the high-resolution (0.05° × 0.05°) mean fields. The CHC’s method for this (Moving Window Regression, or MWR) builds localized regression models using large sets of *in situ* climate normals. Complicated statistical modeling is supported by the fact that there are typically many more available stations to estimate the long-term average conditions than there are to represent variations on a given day or month. The CHC’s MWR process also makes unique use of high-resolution satellite mean fields as predictors. The MWR approach and satellite-means allow the CHC climatologies to perform well in data-sparse regions, and even in regions with complex topography.

Within the CHC’s approach, temporal variations are represented by combinations of *in situ* observations and geostationary satellite-based thermal infrared (TIR) observations. The monthly CHIRTS_max_ product uses a unique cloud-screening process to produce accurate global 2-meter T_max_ anomalies. This accuracy arises through a maximum-compositing process similar to that used in developing gridded vegetation index data sets, such as the Normalized Difference Vegetation Index. Atmospheric water vapor can cause spurious declines in greenness indices. In the case of surface temperatures, partial cloud cover can reduce the temperatures observed by a satellite. In both cases the signature of the contamination is known to suppress the signal. Taking maximum composites over a period of time, therefore, can be used to minimize contamination. For the CHIRTS_max_, this process provides a robust global and very high resolution (0.05°) set of monthly TIR-based temperature anomalies. These anomalies, when combined with the CHC’s high-resolution climatology, provide an accurate and consistent source of estimates, even when there are no nearby weather stations.

Unfortunately, the CHC’s maximum compositing approach cannot work on daily data, because at daily time steps it is difficult to distinguish TIR signal contributions from the land surface and clouds with measurements from only the 11 μm band provided by the GridSat^6^ data set—hence the need for a different approach to disaggregation (Fig. 1b). For this, the CHC uses modern reanalyses. Modern reanalyses use atmospheric models and assimilation schemes to merge vast quantities of information to produce physically based syntheses that provide a complete description of the land and atmosphere. For example, ERA5 uses a four-dimensional assimilation scheme to assimilate satellite radiances from 25 infrared and microwave sources and satellite scatterometer data from four sources. This rich set of data sources provides valuable information about land surface temperatures, soil moisture, atmospheric water vapor, atmospheric air temperatures, precipitation, clouds, and atmospheric circulation anomalies.

This rich set of information, and all the benefits accruing from physically modeling the Earth’s systems, provides an excellent source of information about diurnal temperature variations. At the same time, it should be recognized that the inclusion of these multiple data sources also creates a stream of input data that is heterogeneous in time. For example, many infrared and microwave-based sounders and profilers only appear late in the data record, typically arriving in the late 1990s or early 2000s. Even relatively consistent imagery coming from geostationary satellites can be substantially influenced by inter-satellite calibration issues or orbital changes in any given satellite. Reanalyses that ingest station data, furthermore, face threats related to large shifts in the station data that go into these reanalyses. Both changes in the satellite systems and observation networks can alter the local energy and water budget, potentially introducing spurious random errors.

The four sources of information used in the CHIRTS_max_ contribute in different ways (Fig. 1a,b). The spatial mean fields provide local context. The carefully validated and curated monthly station and TIR temperature anomalies provide a consistent source of climate information, carefully constructed to reduce potential non-stationary systematic errors. The Berkeley Earth organization (www.berkeleyearth.org) was founded in 2012 to collect, quality control, and analyze an integrated set of global air temperature observations. Details on this data set and methods can be found at http://berkeleyearth.org/methodology. Finally, ERA5 reanalysis information is used to disaggregate within a specific month, and greatly reduces any possible issues associated with changes in reanalysis inputs.

### CHIRTS_max_

The monthly CHIRTS_max_ product is the foundational data set from which the CHIRTS-daily products are developed. CHIRTS_max_ combines three components: a high-resolution (0.05° × 0.05°) climatology, interpolated *in situ* temperature anomaly fields, and remotely sensed infrared land surface emissions anomalies based on GridSat^6^ B1 Thermal Infrared geostationary weather satellite observations. Complete details can be found in the CHIRTS_max_ manuscript^1^, though a brief description is provided in this subsection and Fig. 1a for completeness.

There are three components that are combined to create the CHIRTS_max_:

1. CHT_clim_, a high-resolution (0.05° × 0.05°) monthly maximum temperature (T_max_) climatology developed using Moving Window Regression^5^ with FAO station normals, ERA5 long-term average 2-meter temperatures, latitude, longitude, and elevation as predictors.
2. CHIRT_max_, a high-resolution (0.05° × 0.05°) monthly time series of satellite-based T_max_ anomalies.
3. CHTS_max_, a high-resolution (0.05° × 0.05°) monthly time series of interpolated monthly T_max_ anomalies based on Berkeley Earth (http://www.berkeleyearth.org) and Global Telecommunication System T_max_ air observations.

Let $\bar{C}$ denote the long-term average (CHT_clim_). Let *I*′ and *S*′ denote, respectively, the CHIRT_max_ and CHTS_max_ anomalies from their individual long-term means. Then, the final CHIRTS_max_ estimate *T* is a weighted linear combination of these three components, as follows:

$$T=\bar{C}+\alpha I{\prime} +\beta S{\prime} $$

where *α* and *β* are weights that sum to 1 and are derived using the expected variance explained by the CHTS_max_ and CHIRT_max_ estimates. The variance explained by the CHTS_max_ component is based on an empirical covariogram and the distance to the closest station. The variance explained by the CHIRT_max_ component is assumed to be 0.25. The weights *α* and *β* are proportional to these variance values. The final CHIRTS_max_ estimate, therefore, is an adjusted version of the climatology (CHTclim). The adjustment is based upon a weighted combination of satellite-derived estimates of T_max_ anomalies (CHIRT_max_) and interpolation-based estimates of station-observed T_max_ anomalies (CHTS_max_). In data-sparse regions, the satellite-derived anomalies will receive greater weight than their interpolation-based counterparts. Conversely, in regions with high station density, the interpolation-based anomalies will receive the greater weight, effectively leveraging the strengths of both data sources.

### Downscaling ERA5

Daily temperatures from the ERA5 are critical to developing the CHIRTS-daily products, as they define the relative evolution of daily temperatures within a given month. The most apparent limitation to using the ERA5 simulations in tandem with CHIRTS_max_ is the difference in spatial scales between the data products. The spatial scale of CHIRTS_max_ is approximately 5 km by 5 km (0.05° × 0.05°), while that of ERA5 is approximately 25 km by 25 km (0.25° × 0.25°). To bridge this gap and facilitate the merging of the two data products, the ERA5 simulations are downscaled using bilinear interpolation in the Interactive Data Language (IDL^7^) using the CONGRID command. Maximum and minimum temperatures for each day are treated independently in the downscaling procedure. Additionally, there is no explicit treatment of day-to-day temporal dependence. We assume that the inherent temporal autocorrelation is captured by the ERA5 simulations and is preserved in the downscaling routine. We also assume the dependence between maximum and minimum temperatures on a given day is preserved in the downscaling process. Ultimately, the decision to use the ERA5 simulations to disaggregate the monthly CHIRTS_max_ to daily scale is motivated by the latency of the product. Our goal is to provide updates to the CHIRTS-daily product with minimal delay. Collaboration with partners at the National Oceanic and Atmospheric Administration (NOAA) should ensure timely updates to the monthly CHIRTS_max_ product. These updated CHIRTS_max_ products will then be disaggregated with ERA5, providing a much-needed source of information that can be used to monitor extreme temperature conditions. These conditions can have dire impacts on human and livestock health and crops, while also setting the stage for potentially extensive wildfires.

### CHIRTS-daily

To produce the CHIRTS-daily T_max_ (CHIRTS_X_) values, the downscaled ERA5 T_max_ are first translated into anomalies from the monthly ERA5 T_max_ average. These daily T_max_ anomalies are then added to each month’s CHIRTS_max_ value. The resulting CHIRTS_X_ product thus varies on monthly timescales with the CHIRTS_max_ while tracking the day-to-day variations of the ERA5 reanalysis. The ERA5 T_max_ and T_min_ are then used to determine the daily diurnal temperature range (DTR) at each 0.05° pixel. DTR is then used to produce CHIRTS-daily T_min_ (CHIRTS_N_) by subtracting the DTR from CHIRTS_X_.

The steps taken to produce the CHIRTS-daily temperature fields are summarized as follows.

1. Compute the DTR using the downscaled ERA5 fields:

- $$DT{R}_{t}=ERA{5}_{{X}_{t}}-ERA{5}_{{N}_{t}}$$ for $$t=1,\ldots ,T$$

2. Convert the downscaled ERA5 T_max_ to anomalies:

- $$ERA{5}_{{X}_{t}}^{m,anom}=ERA{5}_{{X}_{t}}^{m}-ERA{5}_{X}^{m}$$ for $$t=1,\ldots ,T$$ and $$m=1,\ldots ,M$$

3. Apply the results of Step 2 to the monthly CHIRTSmax values to produce CHIRTS_X_:

- $$CHIRT{S}_{{X}_{t}}^{m}=CHIRT{S}_{max}^{m}+ERA{5}_{{X}_{t}}^{m,anom}$$ for $$t=1,\ldots ,T$$ and $$m=1,\ldots ,M$$

4. Apply the results of Step 1 to CHIRTS_X_ to produce CHIRTS_N_:

- $$CHIRT{S}_{{N}_{t}}=CHIRT{S}_{{X}_{t}}-DT{R}_{t}$$ for $$t=1,\ldots ,T$$

In the above equations, T represents the total number of days in the CHIRTS-daily data record, where (1, 2, 3, …, T-2, T-1, T) represents (Jan 1 1983, Jan 2 1983, Jan 3 1983, …, Dec 29 2016, Dec 30 2016, Dec 31 2016); M represents the total number of months in the CHIRTS_max_ data record, where (1, 2, 3, …, M-2, M-1, M) represents (Jan 1983, Feb 1983, Mar 1983, … Oct 2016, Nov 2016, Dec 2016).

### Ancillary data fields and Heat Index calculation

As a convenience to end users, several ancillary daily variables have been derived from the ERA5 archive and provided at a downscaled 0.05° resolution matching that of CHIRTS-daily. The downscaling procedure used to produce these ancillary data fields is the same used to downscale the ERA5 temperature fields (i.e., CONGRID command in IDL). These data have not received additional validation but are provided to facilitate research for the end users. The ERA5 data set, on which these fields are based, has been widely used and validated. Hourly temperature and dew point temperature from the ERA5 data set are used to estimate relative humidity (RH). These derived RH values, along with CHIRTS-daily, are used to calculate the Heat Index (HI) using the series of equations and rules provided by the National Weather Service (NWS).

Relative humidity (RH) is estimated from temperature and dew point temperature using the Magnus equation^8^, as follows:

$$RH\cong 100\ast exp\left(c\ast b\ast \frac{\left(TD-T\right)}{\left(c+T\right)\ast \left(c+TD\right)}\right)$$

where the constants are defined as b = 17.625 and c = 243.04^8^.

The HI is calculated using the equations provided by the NWS. The main HI equation is a refinement of the multiple regression analysis from a 1990 NWS Technical Attachment (SR 90-23). This regression modeled a set of estimated “apparent temperatures” based on a model of human biophysical thermal temperature equilibria^9^. The following set of equations, referred to as the Rothfusz regression, is abstracted from the NWS website (https://www.wpc.ncep.noaa.gov/html/heatindex_equation.shtml):

$$\begin{array}{lll}HI & = & -42.379+2.04901523\ast T+10.14333127\ast RH-0.22475541\ast T\ast RH\\  &  & -0.00683783\ast {T}^{2}-0.05481717\ast R{H}^{2}+0.00122847\ast {T}^{2}\ast RH\\  &  & +0.00085282\ast T\ast R{H}^{2}-0.00000199\ast {T}^{2}\ast R{H}^{2}\end{array}$$

where *T* is temperature in degrees Fahrenheith (F) and *RH* is relative humidity in percent. *HI* is the heat index expressed as an apparent temperature in degrees F. If the *RH* is less than 13% and the temperature is between 80 and 112 degrees F, then the following adjustment is subtracted from *HI*:

$$AD{J}_{1}=\frac{13-RH}{4}\ast \sqrt{\frac{17-{\rm{abs}}\left(T-95\right)}{17}}$$

If the *RH* is greater than 85% and the temperature is between 80 and 87 degrees F, then the following adjustment is added to *HI*:

$$AD{J}_{2}=\frac{RH-85}{10}\ast \frac{87-T}{5}$$

The Rothfusz regression is not appropriate when conditions of temperature and humidity warrant a heat index value below 80 degrees F. In those cases, we mask and flag the cells where these conditions occurred.

## Data Records

The CHIRTS-daily products are available on the Climate Hazards Center website^10^. To the extent possible under the law, we have waived all copyright and related or neighboring rights to CHIRTS. The data have been assigned to the public domain using the Creative Commons CC0 1.0 Universal waiver. This work is published from the United States. The Climate Hazards Center website also provides access to the validation data used in this paper (http://data.chc.ucsb.edu/products/CHIRTSdaily/v1.0/ValidationData.zip).

At the moment, CHIRTS-daily are available as a climate data record that spans 1983–2016. The 1983 starting point corresponds with the starting point of a reasonably complete global thermal infrared geostationary satellite archive. Future efforts will update the CHIRTS_max_ and CHIRTS-daily on a routine basis. We anticipate that there will be an early release CHIRTS-daily product, available with a delay of approximately 1 month. The final CHIRTS-daily products will be released with an anticipated latency of 2 months.

CHIRTS-daily data are provided in GeoTiff format, though alternate formats may be available upon request. Units are degrees Celsius.

## Technical Validation

### Station screening

To construct the validation data set, we leverage daily station observations from the GHCN and GSOD archives provided by NOAA’s Climate Prediction Center. A stringent and robust station screening process ensures that the validation station data are consistently reported over the CHIRTS_max_ era (1983–2016). Four independent screenings are carried out: one for each source (GHCN and GSOD) and each variable (T_max_ and T_min_). Below, we describe the screening process for GHCN T_max_ stations. This screening process is applied to provide more reliable data for comparison with CHIRTS-daily. The other three data sources (GHCN T_min_, GSOD T_max_, GSOD T_min_) are screened in an identical fashion.

For the GHCN T_max_ screening process, we extract all the daily GHCN maximum temperature station data for the period 1 January 1983 to 31 December 2016, which amounts to ~115 million observations across 17,355 stations. The first step in the screening process is to exclude all stations that have fewer than 2,920 observations (an approximation of 80% reporting for at least 10 years), which leaves ~100 million observations across 12,830 stations. The remaining steps are quantitative in nature, and described in the following rules:

1. Calculate the station median and standard deviation across all days and years for each month.
2. Calculate the z-score for each day, using the following equation:
  $${z}_{t}={x}_{t}-{m}_{i}/{s}_{i},\,i=1,\ldots ,12,$$
  where *z*_*t*_ is the computed z-score for day *t*, *x*_*t*_ is the observation for day *t*, and *m*_*i*_ and *s*_*i*_ are the station median and standard deviation for the *i*^*th*^ month (January = 1, February = 2, etc.).
3. Remove **observations** that satisfy at least one of the following conditions:

- *z*_*t*_ < 4.0
- *z*_*t*_ > 4.5
- *x*_*t*_ − *m*_*i*_ > 20 °C

* These z-score checks (3a and 3b) were intended to screen out potentially mis-coded data. It is common, for example, that data can be recorded unintentionally scaled by a factor of 10. Z-scores beyond ± 4 are extremely uncommon. A larger positive z-score (4.5) was used because we expect climate change-related non-stationarity.

4. Perform a median check for false zero contamination. There are times when the median across all days and years is exactly zero and/or the number of observations that are exactly zero is excessive (~15% of the time). Remove **stations** that meet either of these criteria. Repeat two more times, computing new station medians and standard deviations each time.
5. Finally, remove **stations** that satisfy at least one of the following conditions. Note that *CHTclim*_*i*_ denotes the monthly T_max_ climatology for the *i*^*th*^ month, and *m*_*i*_ again denotes the station median for the *i*^*th*^ month.

- abs(*m*_*i*_ − *CHTclim*_*i*_) > 5 °C
- abs(*m*_*i*_ − *CHTclim*_*i*_)/*s*_*i*_ > 3
- The number of remaining observations is less than <2,920

This leaves 58.3 million GHCN T_max_ observations across 8,587 stations. We then repeat this screening process for GHCN T_min_, and again for GSOD T_max_ and T_min_. What remains are 15,713 T_max_ stations from GHCN (8,587) and GSOD (7,126), with an estimated overlap of 1,612 co-registered stations, and 13,997 T_min_ stations from GHCN (7,747) and GSOD (6,250), with an estimated overlap of 1,390 co-registered stations. We identified a station as potentially co-registered between sources if its coordinates in one source are similar enough to be within 1 km of any set of coordinates in the other source. This method is required, as station identification codes are inconsistent between sources. Note that because the station screening processes are independent of source and variable, some stations will not necessarily be included in both (T_max_ and T_min_) validation data sets.

### CHIRTS_X_ validation results

The CHIRTS_X_ product is validated alongside the PGF^3^ T_max_ product (hereafter PGF_X_ for convenience) using the GHCN and GSOD stations that passed the screening process. To remove any inherent correlation due to the seasonal cycle, all observations in the validation data sets are converted to anomalies at the monthly scale. That is, the monthly mean T_max_ for January is subtracted from all daily January observations, and so on, for all months. This ensures that the mean of the observations at any station will be zero, and that the seasonal harmonics are removed. The same normalization is applied to the CHIRTS_X_ and PGF_X_ products. Each station in the validation data set is then paired with the nearest CHIRTS_X_ and PGF_X_ pixel, using the Euclidean distance from the station coordinates to the pixel centroid. Validation statistics are computed for the hottest three-month period, which is defined using the CHT_clim_ (see Fig. 1c).

Panels A-D in Fig. 2 show the validation statistics for CHIRTS_X_ in the left column and the difference between CHIRTS_X_ and PGF_X_ in the right column (computed as CHIRTS_X_-PGF_X_). CHIRTS_X_ consistently exhibits a stronger agreement with the validation data set than PGF_X_. The prevalence of blue in panel B indicates that CHIRTS_X_ has consistently higher correlations with the stations than PGF_X_—the global average is 0.17. Similarly, the abundance of red in panel D indicates CHIRTS_X_ has consistently lower mean absolute error (MAE) than PGF_X_—the global average is −0.77 °C. Table 1 summarizes these validation statistics by region/continent, which further illustrates the skill of CHIRTS_X_.

**Table 1:** Validation statistics for maximum temperature products.

T_max_	Correlation		Mean Absolute Error	
	CHIRTS_X_	PGF_X_	CHIRTS_X_	PGF_X_
Global	0.84	0.66	1.3	2.2
N. America	0.80	0.63	1.6	2.4
S. America	0.72	0.45	1.3	2.2
Africa	0.81	0.57	1.1	2.0
Eurasia	0.91	0.74	1.0	1.9
Australia	0.92	0.75	1.0	2.1

### CHIRTS_N_ validation results

We next compare the performance of CHIRTS_N_ to that of the PGF T_min_ product (PGF_N_ for convenience) for the same hottest 3-month period as the CHIRTS_X_ validation (Fig. 1c). We did this in order to maintain consistency with the CHIRTS_X_ validation, and to focus our validation on performance during the hottest times of the year.

We convert the T_min_ observations from GHCN and GSOD to monthly anomalies to remove inherent correlation. Each station is paired with the nearest CHIRTS_N_ and PGF_N_ pixels, and validation statistics are computed. Panels E-H in Fig. 2 show the validation statistics for CHIRTS_N_ in the left column and the difference between CHIRTS_N_ and PGF_N_ in the right column (computed as CHIRTS_N_-PGF_N_). Consistent with the CHIRTS_X_ validation, the CHIRTS_N_ correlations are consistently higher and the MAE are consistently lower than that of PGF_N_. The global average difference in correlations between CHIRTS_N_ and PGF_N_ is 0.09; the global average difference in MAE is −0.37°. Table 2 summarizes these validation statistics by region/continent.

**Table 2:** Validation statistics for minimum temperature products.

T_min_	Correlation		Mean Absolute Error	
	CHIRTS_N_	PGF_N_	CHIRTS_N_	PGF_N_
Global	0.75	0.65	1.4	1.8
N. America	0.78	0.71	1.4	1.8
S. America	0.64	0.48	1.2	1.6
Africa	0.67	0.46	1.2	1.8
Eurasia	0.78	0.67	1.3	1.6
Australia	0.60	0.51	1.9	2.3

### Mean bias error

It is important to acknowledge the fact that we are validating the CHIRTS-daily data series in anomaly space. It is therefore crucial to quantify potential mean bias error in these data sets. To this end, for every GHCN and GSOD station and every month, we compute the station climatologies and compare these values to the mean of the CHIRTS_X_ and PGF_X_ for the same collection of days. We compute these differences as CHIRTS_X_-station and PGF_X_-station, thus a positive (negative) value indicates a warm (cool) bias in the data product. This produces 12 difference values at every validation station, which we then collapse (average) into a mean bias error statistic. Table 3 summarizes the mean bias error globally and by region. These results indicate that CHIRTS_X_ is much less biased from a global perspective, and consistently better for many regions because of its low bias and good performance in data-sparse regions. However, CHIRTS_X_ exhibits a significant positive bias for Australia that is not present in PGF_X_.

**Table 3:** Mean bias error in degrees Celsius for the maximum temperature validation data set.

T_max_	Mean Bias Error (°C)	
	CHIRTS_X_	PGF_X_
Global	0.12	−0.24
N. America	0.01	−0.06
S. America	−0.16	−0.30
Africa	0.11	−0.23
Eurasia	0.21	−0.43
Australia	0.39	0.00

Positive (negative) values indicate the data product is consistently warmer (cooler) than observations.

### Performance in data-sparse regions

What distinguishes the CHIRTS_max_ from other gridded products is its use of remotely sensed thermal infrared temperatures. Where large gaps in station networks exist, the resulting interpolation-based gridded products are highly uncertain. Furthermore, station-based gridded products tend to revert to climatology as the distance to the nearest *in situ* observation increases. This likely underestimates the variance in data-sparse regions. It follows that the performance of CHIRTS_X_ in data-sparse regions is bolstered by remotely sensed observations, which inherently makes it more reliable in data-sparse regions.

To verify this assumption, we analyze the performance of CHIRTS_X_ and PGF_X_ as a function of distance from the nearest station included in the monthly CRU data product. We focus on CRU stations because the CRU product is widely considered the gold standard of gridded climate products. Additionally, the CRU gridded products are used to bias correct the PGF in its development workflow. Empirical covariograms are estimated for each product at a set of sequential distances bins, calculated as R^2^ = 1 − MSE/SS, where MSE is the Mean Squared Error, and SS is the Sum of Squares of the station anomalies.

Figure 3a shows the expected performance for the warmest 3 months, expressed as R^2^ values for CHIRTS_X_ and PGF_X_. When both products are assessed at a station location (i.e., at a distance of zero), CHIRTS_X_ has an R^2^ that is about 0.20 greater than that of PGF_X_. At about 300 km, the performance of both products begins to decline at the same rate. However, at about 650 km, the performance of PGF_X_ begins a slow, oscillating decline. The performance of CHIRTS_X_ hovers around R^2^ = 0.63, while that of PGF_X_ is closer to R^2^ = 0.15. At 1500 km, the performance of PGF_X_ drops to R^2^ = 0, while CHIRTS_X_ maintains a value of R^2^ = 0.50. It can be clearly stated that leveraging the thermal infrared temperatures in the CHIRTS_max_ product effectively enhances CHIRTS_X_ in data-sparse regions.

Figure 3b shows a map of the expected variance explained by CHIRTS_X_ based on the distribution of stations in the Berkeley Earth archive for January 2016. Figure 3c shows the same for PGF_X_ based on the distribution of stations in the CRU archive for January 2016. The performance of CHIRTS_X_ is bolstered by not only the thermal infrared background of the monthly CHIRT_max_—a satellite-only component of the monthly CHIRTS_max_, which was shown to have a correlation of about 0.80 with *in situ* observations globally^1^—but also a much denser distribution of stations than PGF_X_, which results in a considerably more reliable data set.

## Usage Notes

This section provides two examples of usage case studies, representing typical applications for the CHIRTS-daily maximum temperatures. The first application uses a 40.6 °C temperature threshold. This threshold, often used in human health studies, represents a temperature level at which the human body often has difficulty maintaining adequate cooling of internal organs. The second application uses a threshold of 30 °C, a common threshold used to identify agricultural heat stress. The first case study is global. The second case study focuses on Ethiopia, expanding on supplemental material provided in the CHIRTS_max_ manuscript^1^.

**Fig. 4:**
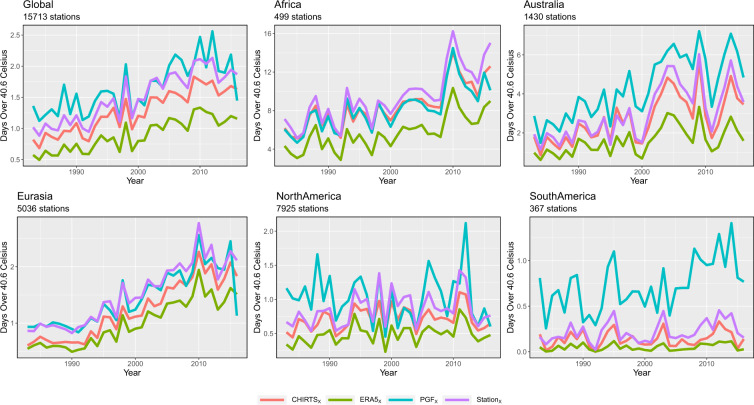
Time series of the average number of days over 40.6 °C for stations in various regions based on the observations (Stations), CHIRTS_X_, ERA5_X_, and PGF_X_ data sets.

Figure 4 presents a comparison of the daily station data, CHIRTS-daily, ERA5, and PGF archives, for the warmest 3 months. The CHIRTS-daily, ERA5, and PGF values have been extracted at the station locations, supporting a one-to-one comparison. What is quite striking in all the regions examined is that ERA5 dramatically underestimates the number of hot days in all regions. The PGF archive, on the other hand, substantially overestimates in Australia and South America. The CHIRTS-daily, in all cases, tracks the station-based estimates very closely. Note that the daily GHCN and GSOD data likely contributed to the monthly Berkeley Earth data used in the monthly CHIRTS_max_, so Fig. 4 should not be interpreted as an independent validation study, which is beyond the scope of this data descriptor. Nevertheless, CHIRTS-daily does appear to be fit-for-purpose for analyzing trends in temperature extremes, especially in data-sparse regions. It should be noted that Africa stands out as a region with large increases in the number of very hot days (Table 4). While all the gridded data sets underestimate the station data change estimate (+5.7 days), the CHIRTS-daily estimate was the closest (+4.7 days). More analysis of the spatial pattern and health hazards associated with these large increases appears warranted.

**Table 4:** Change in the mean number of hot days (over 40.6 °C) between 2007–2016 and 1983–1992.

	CHIRTS	ERA5	PGF	Station
Global	+0.77	+0.55	+0.74	+0.88
N. America	+0.13	+0.14	−0.01	+0.20
S. America	+0.04	+0.03	+0.45	+0.13
Africa	+4.70	+3.40	+4.30	+5.70
Eurasia	+1.20	+0.92	+1.00	+1.20
Australia	+1.80	+0.88	+2.90	+2.20

We next turn to a typical agro-climatic risk assessment. Building on results presented in the supplemental material of the CHIRTS_max_ manuscript^1^, this case study focuses on July T_max_ temperatures in the Amhara province of northern Ethiopia. El Niño-related rainfall deficits in this region^11^ contributed to “the worst drought in 50 years” and led to widespread crop failures that helped push approximately 11 million people into crisis levels of food insecurity.

**Fig. 5:**
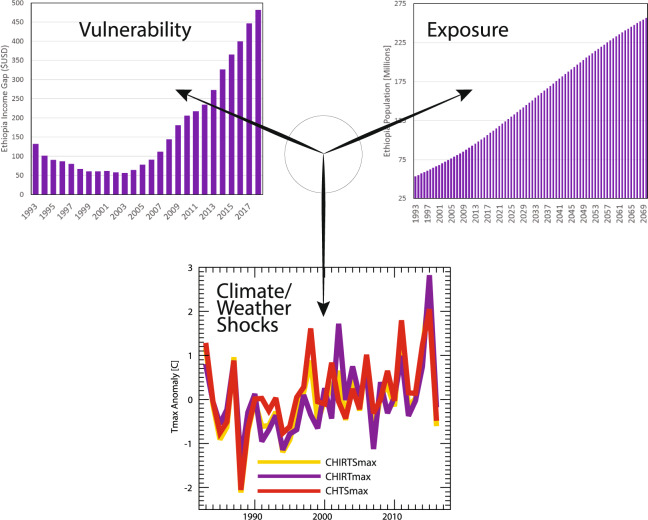
A schematic representation of climate hazard dimensions for Ethiopia. Climate/weather shocks are represented by monthly July T_max_ anomalies for the satellite-only CHIRT_max_ data set, the station-only CHTS_max_ archive, and the blended satellite-station CHIRTS_max_ product. The vulnerability to price and crop production shocks is represented by the income gap between the poorest 20% of Ethiopians and the median income. Exposure is represented by national population estimates.

These low rainfall levels were also accompanied by exceptionally warm July air temperatures (Fig. 5). The unique nature of the monthly CHIRTS_max_ archive provides independent satellite-only temperature estimates (CHIRT_max_) and station-only temperature estimates (CHTS_max_), as well as the blended “best estimate” CHIRTS_max_ product. Humanitarian relief agencies often use a convergence-of-evidence approach to guide drought assessments. The fact that the independent CHIRT_max_ and CHTS_max_ archives both indicated historically extreme air temperatures provides convergent evidence of severe potential crop stress.

Climate hazards typically involve this type of climate shock with underlying vulnerability and exposure—Fig. 5 represents this schematically. Vulnerability is represented by the gap between the median and 20^th^ percentile World Bank per capita incomes. Despite increases in agricultural productivity, the number of extremely food insecure Ethiopians has increased. This increase may be due to a series of recent climate shocks, combined with an increased price-spike vulnerability, for poorer Ethiopians (http://blog.chc.ucsb.edu/?p=634). Figure 5 represents the third dimension of climate hazards—exposure—using United Nation estimates of the Ethiopian population. Between 1993 and 2070, Ethiopia may experience a five-fold increase in population, with the number of Ethiopians increasing from about 50 to 250 million people.

**Fig. 6:**
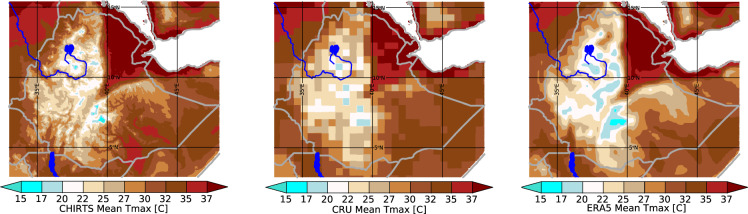
A comparison of July T_max_ 1983–2016 mean fields for Ethiopia. From left to right, the panels show means derived from CHIRTS_max_, CRU, and ERA5 data sets.

While the ~+2.5 °C temperature anomaly shown in Fig. 5 appears concerning, it is difficult to interpret from an agronomic perspective. Plants typically respond to the actual temperature values. In colder areas, crops may actually benefit from warmer temperatures. In hot areas, temperature increases can result in increased wilting and moisture loss. This dependence means that an accurate background climatology can improve the utility of climate hazard assessments. Figure 6 shows long-term July T_max_ values averaged over 1983–2016 for the CHIRTS-daily, CRU, and ERA5 data sets. The high-resolution CHIRTS-daily product clearly represents Ethiopia’s complex orographic influences. We find some of the steepest temperature gradients on Earth, with highland areas having mean maximum temperatures ranging from 15 to 20 °C, while nearby lowland areas may have mean values of greater than 37 °C. The Moving Window Regression^5^ modeling process used to construct the CHIRTS background climatology uses satellite TIR mean fields as a predictor, and these fields help capture these complex gradients. These patterns are not captured well by the coarse CRU product. The physically based ERA5 reanalysis captures the overall pattern with reasonable fidelity, but many fine details are missed. Furthermore, as noted above in the global case study, there appears to be a consistent tendency to underestimate the magnitude of the mean maximum temperatures, making this an inappropriate product for estimating the hazards associated with temperature extremes.

**Fig. 7:**
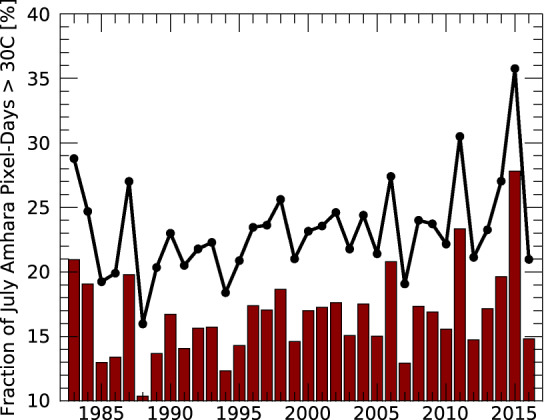
Time series of observed and projected frequency of agricultural heat stress for Ethiopia’s Amhara province in July. The barplot shows the frequency based on CHIRTS-daily maximum temperatures. A value of 25 means that 25% of the day-pixels had maximum temperatures exceeding 30 °C. The black line with filled circles represents a plausible climate change scenario in which the temperatures have been increased by +1.3 °C.

For agricultural impact assessments, days over a 30 °C threshold is a common metric of heat stress. The bar plot shown in Fig. 7 shows a time series of this metric for consecutive Julys in the Amhara province of Ethiopia—a critical crop-growing region strongly impacted in 2015. This time series gives us a meaningful basis for assessing agricultural temperature-related impacts. First, note that this time series is quite variable. In some years, only 10% of the pixel-days exceeded 30 °C. In 2015, on the other hand, almost 30% of the pixel-days exceeded 30 °C. This suggests widespread temperature impacts that negatively influenced crop production—capturing such impacts from space may be quite valuable. Current crop water accounting approaches, such as the Water Requirement Satisfaction Index metric (https://earlywarning.usgs.gov/fews/product/126), did not adequately capture the magnitude of 2015 agricultural yield anomalies in Amhara.

We conclude with a brief 2070 climate change “projection” for Amhara, based on a perturbed version of the observed CHIRTS-daily data set. Note that our goal here is to simply provide a representative usage case, not to perform a detailed climate change assessment. In general, it is not unreasonable to assume that future air temperatures may be represented by current air temperatures (CUR) perturbed by a smoothly varying set of increases (DELTA). Furthermore, let CUR be a data set with high spatial and temporal resolution, like CHIRTS-daily. DELTA may be derived from climate models such as those represented in the Phase 5 or 6 Coupled Model Intercomparison Projects. Here, we use a single value of +1.3 °C, which is the change in the Phase 5 multi-model ensemble T_max_ average, for Amhara in July, between 2070 and 2020, based on the 6 Wm^−2^ Representative Concentration Pathway. Perturbing our data set with this value and recalculating the fraction of pixel-days warmer than 30 °C results in the black line and filled circles in Fig. 7. By 2070, Ethiopia is likely to have a population of ~250 million people (Fig. 4), and the average heat stress fraction may be around 23%, a level exceeded in only the most extreme months in the observational record.

## Data Availability

The CHIRTS-daily data products are derived using approximately 500 lines of code written in the Interactive Data Language. While not written as a portable library or toolset, access to the code is not restricted, and it is available upon request.
